# The health impacts of extractive industry transnational corporations: a study of Rio Tinto in Australia and Southern Africa

**DOI:** 10.1186/s12992-019-0453-2

**Published:** 2019-02-19

**Authors:** Julia Anaf, Frances Baum, Matt Fisher, Leslie London

**Affiliations:** 1 Southgate Institute for Health, Society and Equity, Flinders University, GPO Box 2001, Adelaide, SA 5001 Australia; 2 Department of Public Health and Family Medicine, University of Cape Town, Cape Town, South Africa

**Keywords:** Extractive industry, Globalization, Health equity, Transnational Corporations

## Abstract

**Background:**

Operations of transnational corporations (TNCs) affect population health through production methods, shaping social determinants of health, or by influencing regulation of their activities. Research on community exposures to TNC practices and policies has been limited. Our research on extractive industries examined Rio Tinto in Australia and Southern Africa to test methods for assessing the health impacts of corporates in high and middle income jurisdictions with different regulatory frameworks.

**Methods:**

We adapted existing Health Impact Assessment methods. Data identifying potential impacts were sourced through media analysis, document analysis, company literature and semi-structured interviews. The data were mapped against a corporate health impact assessment framework (CHIA) which included Rio Tinto’s political and business practices; productions; and workforce, social, environmental and economic conditions.

**Results:**

Both positive and detrimental aspects of Rio Tinto’s operations were identified. Requirements imposed by Rio Tinto on its global supply chain are likely to have positive health impacts for workers. However, political lobbying and membership of representative organisations can influence government policy in ways that are unfavourable to health and equity. Positive impacts include provision of direct employment under decent working conditions, but countered by an increase in precariousness of employment. Commitments to upholding sustainable development principles are undermined by limited site remediation and other environmental impacts. Positive contributions are made to national and local economies but then undermined by business strategies that include tax minimisation.

**Conclusion:**

Our study confirmed that it is possible to undertake a CHIA on an extractive industry TNC. The different methods provided sufficient information to understand the need to strengthen regulations that are conducive to health; the opportunity for Rio Tinto to extend corporate responsibility initiatives and support their social licence to operate; and for civil society actors to inform their advocacy towards improving health and equity outcomes from TNC operations.

## Background

### Introduction

Transnational corporations (TNCs) are incorporated or unincorporated enterprises comprising parent enterprises and their foreign affiliates. A parent enterprise controls the assets of other entities in countries other than its home country; usually by owning an equity capital stake [1]. TNC revenues now exceed those of many national governments [2]. It is estimated that TNCs directly employ 4 % of workers in developed countries and 12% in developing countries; with approximately 60,000 parent TNCs, and an estimated 500,000 subsidiaries worldwide [3]. The legal status granted to corporations in the USA, and to differing degrees in other countries, includes the right of legal ‘personhood’, limited shareholder liability, an unlimited lifespan, the right to sue and be sued, and the right to own stock [4, 5].

As major players in global value and network chains [6] TNC operations include both products and practices that affect population health [7]. An expanding body of research is being conducted on the operations of TNCs in relation to health in many sectors, including food and beverage [8–10], extractive, pharmaceutical, and tobacco [11–13]. TNCs directly influence the social determinants of health (SDH) such as employment and living conditions, income, education, environmental conditions, food environments and social support [10, 14]; and also influence the policy structures and decisions that determine inequalities in the distribution of conditions favourable or unfavourable to health [15]. They thereby contribute to health inequities within or between nations [14]. The role of national governments to cooperate to regulate TNC operations globally, or to regulate operations within their own jurisdictions, is inhibited by a ‘free trade’ environment in which TNCs are able to shift investment between countries [16]. Mining corporations have used the environment of free trade to offshore their operations and thereby circumvent domestic opposition to their activities. Free trade agreements have allowed mining companies to expand their activities, and seek recourse in international tribunals to override local government sovereignty [17]. Global supply chains may also undermine work, health and safety regulation when corporations operate in developing countries where regulation is often weak or absent [18].

Extractive industry TNCs take raw materials from the earth including metals, minerals and aggregates, oil and gas (Business Directory.com). Mining is a major component of extractive industries. Approximately 3.5 billion people live in countries rich in minerals, oil or gas, and these resources play a significant role in the economies of 81 of 195 countries worldwide; accounting for a quarter of global GDP [19]. Africa alone is home to approximately 30% of the world’s mineral reserves, 10% of the world’s oil, and approximately 8 % of the world’s natural gas [19].

Under good governance arrangements that respect rights, community needs and the environment, extractive industry revenues can help to reduce poverty and boost shared prosperity [17] through increased employment opportunities, improved working conditions, and infrastructure spending [20]. National taxation revenue from extractive industries potentially augments outlays for health and social services [21]. Large TNCs may deliver needed services even when the state is absent or delinquent as happened with retroviral services for HIV-infected miners during a period of AIDS denialism by the state in South Africa in the late 1990s / early 2000s [22]. Some extractive industry TNCs commit to corporate social responsibility programs; assessing their health, environmental and social impacts and benchmarking these against competitors [23].

However, evidence also shows adverse social, environmental and health impacts arising from extractive industry practices [17]. Negative environmental impacts may include soil, water and air pollution; deforestation and erosion; dumping of hazardous wastes; and increased coastal erosion and desertification [13, 24]. Impacts on air quality and agricultural production may create health risks for populations living adjacent to mining operations, leading to environmental refugees [17]. Mining operations can expose workers to major physical, chemical, biological, ergonomic, psychosocial, and occupational health hazards [25]. Downing notes a ‘resettlement effect’ on communities living near mining operations; characterised by loss of assets (homes, communities, and productive land), ‘destruction of cultural sites, diminution of cultural identity, and disruption of social structures, networks and mutual help mechanisms’ ([26] p. 3). Available data indicate that the spread of toxic chemicals in the environment is a public health crisis; causing cancer, sterility, respiratory problems, chemical hypersensitivity, birth defects, and nervous system and vital organ dysfunctions [13]. Most toxic wastes are externalities of industrial production in which the costs of adverse health impacts are shifted to the wider community [13].

Despite the impact of extractive TNCs there has been little research to assess their overall population health and equity impact. Accumulated evidence on SDH provides the framework for our understanding of TNC health impacts [14] recognising that TNC practices may directly affect the living conditions and access to services that affect health, and may contribute to the political and socioeconomic inequalities that cause health inequities [27]. In this paper we apply a corporate health impact assessment (CHIA) framework to assess the health impacts of one extractive industry (predominantly mining) TNC, Rio Tinto, in Australia, South Africa and Namibia; three countries with different regulatory structures and significant differences in population health [28]. Our framework is focused on regulatory structures, corporate practices and products, and direct impacts on daily living conditions [29] and complements the 2018 framework by Schrecker et al. [30]. The latter framework links the historical context of global extraction and pathways to ill health and the consequences for SDH. Schrecker et al. also include an analysis of the political economy of extractivism, focusing on the societal structures, processes, and power relationships that drive and enable extraction [30].

## Methods

### Step 1: Adapting HIA methods to assess extractive TNC operations

#### Health impact assessment

Health Impact Assessment (HIA) provides a structured methodology for systematically identifying and assessing potential health impacts of governmental or organisational policy or practice changes and developing policy recommendations to minimise negative, and enhance positive, impacts [31–33]. HIAs can be used prospectively to assess likely impacts in the future, or retrospectively to assess impacts of past events, predict future impacts and inform decision-making [34].

**Fig. 1:**
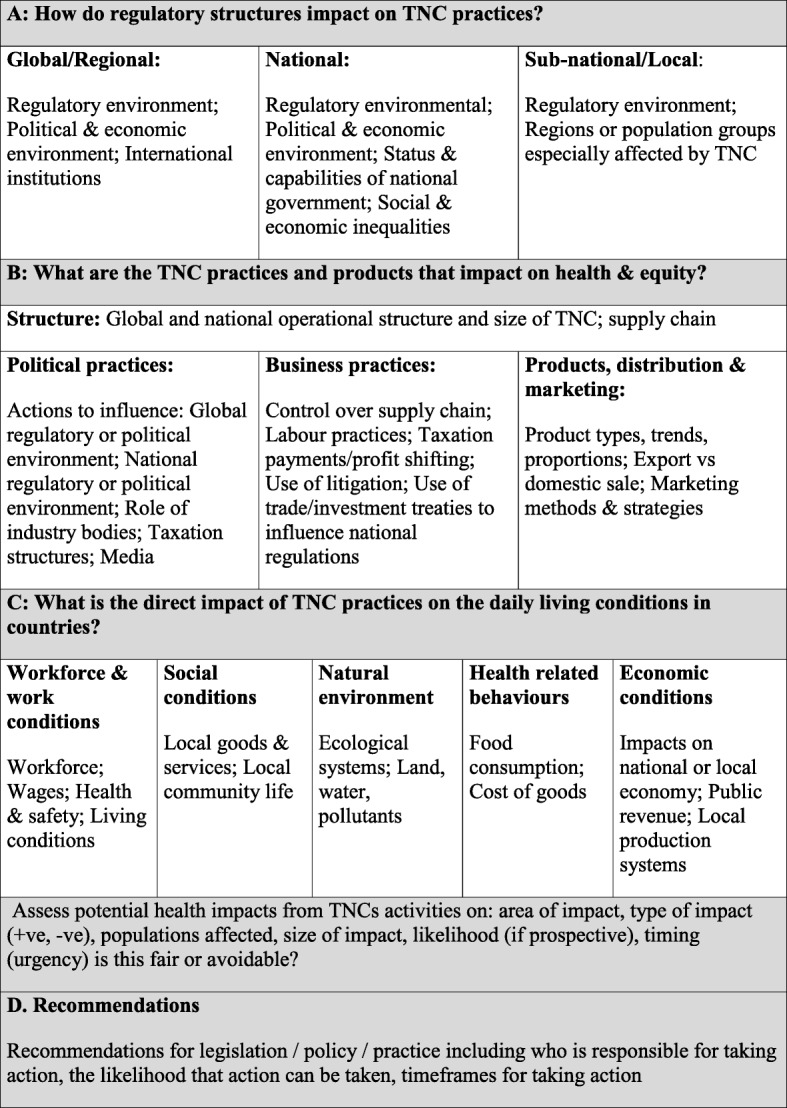
Corporate Health Impact Assessment Framework. Conceptual pathways of the health impacts of Transnational Corporations (TNCs) on population health

In 2015 the authors contributed to development of a Corporate Health Impact Assessment (CHIA) Framework (Fig. 1) [29, 35]; applied in this paper to identify and assess positive and detrimental health impacts from Rio Tinto’s corporate practices.

#### Selecting the extractive industry TNC

Our rationale for choosing the extractive sector was that it has significant impacts on health [25]. We chose Australia and Southern Africa (South Africa and Namibia) to explore differences between Rio Tinto operations and health impacts in high and middle-income jurisdictions with different regulatory and socioeconomic environments. We sought an understanding of how regulation can reduce negative health impacts. Rio Tinto operates in each of these jurisdictions and author (LL) is based in South Africa.

### Step 2: Identifying potential impacts

The CHIA identification stage included profiling the population groups affected by the TNC and collecting information to identify those most likely to experience health impacts [36]. Factors understood to have positive and negative impacts on physical and mental health outcomes, relevant information relating to Rio Tinto’s global operations within Australia and Southern Africa, and regulatory and demographic data were collected and mapped against the three levels of the CHIA framework. We chose not to use the category ‘health-related behaviours’ as no major impacts were identified that related to this category. We considered the global context in which Rio Tinto operates, and how this context shapes its practices in different countries. Our research focussed on assessment of the health impacts relating to operations in Australia and Southern Africa, and a comparison between the two. Health impact was assessed in terms of the effects of Rio Tinto corporate practices on social determinants of health, with attention to impacts on health inequities within or between the selected jurisdictions.

Data were sourced from documents, websites and list-servers, Rio Tinto’s corporate literature, media items, and semi-structured interviews. The original data included approximately 275 items which were added to by later searches. We undertook a detailed description of each of the main mining operations in each jurisdiction. This paper provides an overview of key issues we identified, contextualised by excerpts of interviews with civil society actors.

#### Documents and websites

A keyword search of the Flinders University library holdings and Google Scholar was undertaken for the period 2011–2016. The websites of key civil society activist groups monitoring the operations of TNCs were checked for any references to Rio Tinto. Global data on the political, economic and regulatory context for Rio Tinto’s activities were collected, including information on regulatory institutions, guidelines and standards for multinational enterprises, industry representative organisations, global unions, and voluntary reporting initiatives. At the national and sub-national level information relating to Rio Tinto’s operations in Australia and Southern Africa, and data on work health and safety, employment and wages were collected.

Items reviewed from the Rio Tinto’s website included annual reports, sustainable development reports, global and national taxation payments, media releases, and data from selected Australian and Southern African production sites. Other literature covered Rio Tinto’s voluntary reporting initiatives.

#### Media items

The Factiva database was accessed to identify all news sources under search headings relating to Rio Tinto’s industrial, economic and political news items for Australia and South Africa between 2012 and 2016. The Proquest International Australian and Zealand Newsstream database was searched for newspaper reports relating to Australia and South Africa for the same period. Web based news compilations concerned with mining operations archived between 2014 and 2016 augmented the data [37, 38].

#### Semi-structured interviews

Eleven semi-structured interviews were conducted to gain perspectives on Rio Tinto’s operations in Australia and southern Africa, including from Rio Tinto and / or the mining industry sector, and from civil society actors and campaigners monitoring Rio Tinto’s operations across different mining operations. We invited Rio Tinto and key extractive industry representatives to participate in the research but all declined. The barriers to engaging political and business elites as participants in academic research have been documented [39, 40]. Civil society and NGO actors were also invited to participate. Australian participants were identified through purposive and snowball sampling based on high profile civil society and union campaigns in different states. In Southern Africa respondents were identified through international union information and snowball sampling facilitated by contacts in South Africa with an understanding of Rio Tinto’s operations. Interview schedules were designed to elicit responses in the areas of health impact identified across the CHIA framework. Each potential respondent was emailed a consent form, and an invitation to participate and project information sheet. The 11 interviews were conducted via telephone or Skype and transcribed by professional transcription services.

Industriall Union represents 50 million workers in 140 countries in the mining, energy and manufacturing sectors and challenges the power of multinational companies and negotiates with them on a global level. Industriall Union has been active against negative practices by Rio Tinto across different jurisdictions including Namibia and South Africa in respect of precarious employment and other issues and has been a significant source of data for the research [41].

### Step 3: Assessment of impacts

The purpose of the CHIA assessment stage is critically to synthesise and assess the information collected during the identification stage, to help prioritise health impacts [36]. Information from documents and websites, media items and transcribed interviews were imported into NVivo qualitative data analysis software and coded against the three levels of CHIA framework shown in Fig. 1. The areas of health impact in each jurisdiction were coded for positive and detrimental health impacts and aspects of corporate activity. Fortnightly team meetings were held to discuss the progress of the research including coding, emerging themes, and the scope and focus of the comparative analysis of Rio Tinto’s operations within and between the three jurisdictions.

## Results

### Rio Tinto’s global operations

Rio Tinto was formed in 1873 and operates in 35 countries across six continents. It is a dual listed, UK and Australian entity managed in the UK. It is a ‘parent’ company of many subsidiaries through strategic acquisitions, and engages in ongoing divestment and acquisition of assets [41, 42]. Rio Tinto has approximately 200,000 shareholders, and 47,000 staff including approximately 25,000 in Australasia and 5000 in Africa [43]. These figures include the Group’s share of joint ventures and associates. Industriall Union states that Rio Tinto does not report its large numbers of sub-contracted workers [44], meaning the total number of workers is unclear.

Rio Tinto’s business is diversified across five groups: iron ore, copper, energy (coal and uranium), diamonds and minerals [45]. Globally, in 2017 the Group’s direct economic contribution was US$41.8 billion including US$27.7 billion in payments to employees, governments, and returns to capital. In 2017 it invested US$176 million across approximately 1300 programmes covering the health, education, housing, environmental protection and agricultural and business development sectors [43, 46]. This represents 0.44% of the corporation’s US$40 billion sales revenue [47]. In 2017 Rio Tinto paid US$5.1 billion in taxes (corporate taxes, royalties, fees, property taxes, employment taxes and irrecoverable indirect taxes borne by the corporation) globally [48].

Rio Tinto states ‘All our sites must have a complaints, disputes and grievance mechanism that meets the UN Guiding Principles on Business and Human Rights criteria’ [49, 50] and rules set by the World Trade Organisation, the International Labour Organisation and other international institutions regulating work health and safety. These Guiding Principles recognise states’ existing obligations to respect, protect and fulfil human rights and fundamental freedoms. Business enterprises, as specialised arms of society are also expected to comply with all relevant laws and to respect human rights. Rights and obligations must be matched with appropriate and effective remedies when breached. Importantly, the Guiding Principles apply to all states, and to business enterprises, both transnational and others, irrespective of size, sector, location, ownership and structure.

However, the 2012 report of the African Commission’s Working Group on Indigenous populations ([24] p. 66) states that while Rio Tinto makes specific mention of free, prior and informed consent (FPIC) in its community agreements, based on the UN Guiding Principles, it does not make binding commitments to achieve FPIC. Rio Tinto is also a signatory to several voluntary reporting initiatives which are proxies for formal regulations. It operates without any international agreement on taxation obligations, as none has been mandated.

### Rio Tinto’s political practices

Rio Tinto is a member of the Australian Chamber of Commerce, which is part of the International Business Industry Advisory Committee [51] to the Organisation for Economic Co-operation and Development (OECD) [52]. It is a member of the Minerals Council of Australia (MCA) and Business Council of Australia (BCA), and is represented on both boards and respective committees [53, 54]. The MCA is the peak body of Australia’s extractive industry sector [55]. The BCA provides a forum for businesses to directly contribute to public policy debates [56]. Rio Tinto is also a member of the mining industry employer organisation, the Chamber of Mines in South Africa and Namibia [57]. In 2017 the corporation retained the Rio Tinto Lobby Group and Smith-Free lobbyists to lobby the US government on regulation of environmental issues, natural resources, taxes, trade and federal appropriations [58]. Rio Tinto’s Political Action Committee donated US $63,000 to political actors in the 2016 US election [59]. The corporation is a member of the World Coal Association which is an accredited observer to the United Nations Framework Convention on Climate Change (UNFCCC) [60], where it lobbies to undermine renewable energy policies [61]. Rio Tinto has approximately 500 controlled entities, nine of which are resident in tax havens, [43].

### Rio Tinto’s business practices

Rio Tinto incorporates major supply chains with products that require large rail and shipping infrastructure. In 2017 Rio Tinto’s global supply chain included 31,000 suppliers to whom it paid US$14.1 billion for goods and services, governed under a new Supplier Code of Conduct [62]. This code details the expectations of suppliers, their subsidiaries and sub-contractors, with provisions including avoidance of forced, compulsory or child labour, promoting humane treatment, and respecting workers’ rights to join trade unions [63]. A confidential and independently operated multilingual ‘whistleblowing’ service is available to Rio Tinto employees, contractors, suppliers and customers [64]. Rio Tinto has also signed a Slavery and Human Trafficking statement which meets the requirements of the UK Modern Slavery Act 2015 [65, 66]. Compliance is particularly critical for operations in South Africa where the population living in some form of slavery is approximately 25 times larger than in Australia [67].

#### Corporate social responsibility

Rio Tinto is a signatory to a range of corporate social responsibility mechanisms including the Corporate Responsibility Index, Global Reporting Initiative, London Benchmarking Group, FTSE4Good Index, Carbon Disclosure Project, Extractive Industries Transparency Initiative, and the UN Global Compact which encourages businesses to adopt and report on sustainable and socially responsible policies [68]. However, corporate social responsibility has been criticised as a means for corporations to deflect the threat of direct regulation and protect commercial interests [69]. Within the scope of their operations, TNCs can be simultaneously socially responsible and irresponsible [70]. Voluntary codes have little public accountability, are difficult to reinforce, and rely on negative publicity. They also divert attention away from legal compliance, and third party monitoring by NGOs is of limited effectiveness [71]. Research on Rio Tinto’s corporate social responsibility commitments arguably reveals that it departs from these in remote regions in developing nations with poorer governance structures and corruption amongst officials [24, 72].

### Rio Tinto’s Australian, South African, and Namibian operations

Rio Tinto works in different socioeconomic contexts ranging from high income and strongly democratic countries to those with much lower incomes and weaker democratic regimes. Table 1 presents the basic demographic data for Australia, a high income country, and South Africa and Namibia, which are both middle income jurisdictions, to provide insights into the comparative operating contexts.

**Table 1:** Comparative demographic data Australia, South Africa and Namibia

Demographic domain	Australia	South Africa	Namibia
Population (million)	23.9	53.5	2.5
Life expectancy (OECD average 80.9 years)	82.4	57.2	64
Unemployment rate	6.1	26.7	28.1
Income inequality -Gini coefficient (OECD average 0.31)	0.337	0.634	0.572
Relative poverty rate (OECD average 11.0)	12.8	53.8	45.7
GDP per capita (000 $USD PPP) (OECD average 41.2)	46.7	13.7	9
Tertiary education 25–64 years (OECD average 35.7)	42.9	6.4	< 10
Number of International Labor Organization (ILO) Conventions ratified	58	54	11
a) Fundamental	a) 7 of 8	a) 8 of 8	a) 8 of 8
b) Governance	b) 3 of 4	b) 2 of 4	b) 1 of 4
c) Technical	c) 48 of 177	c)17 of 177	c) 2 of 177
Transparency International Corruption Perceptions Index 2017:			
Rank by country 1–89	13	71	53
Corruption Perception Index	(CPI - 77)	(CPI - 43)	(CPI - 51)
World Bank: *Regulatory Quality* Perceptions of government ability to formulate and implement sound policies and regulations (2016 percentile rank 0–100)	97.60	62.02	49.52
World Bank: *Government Effectiveness* Perceptions of quality of public / civil services and degree of independence from political pressures, quality of policy formulation / implementation, and credibility of government commitment. 2016 percentile rank 0–100	92.31	64.90	60.10
World Bank: *Voice / Accountability* Perceptions of the extent to which a country’s citizens are able to participate in selecting their government, as well as freedom of expression and association and a free media 2016 percentile rank 0–100	94.09	67.98	66.50
World Bank: *Political stability and absence of violence* Perceptions of the likelihood of political instability and/or politically motivated violence including terrorism 2016 percentile rank 0–100	81.90	42.38	70.00
World Bank: *Rule of law* Perceptions of the extent to which agents have confidence in and abide by the rules, quality of contract enforcement, property rights, the police, courts, and the likelihood of crime and violence 2016 percentile rank 0–100	95.19	58.17	64.42
World Bank: *Control of corruption* Perception of the extent to which public power is exercised for private gain including corruption and ‘capture’ by private interests 2016 percentile rank 0–100	93.27	65.87	64.42
Average Salary 2018 (world ranking):	US$88,275 [160]	US$47,046 [161]	US$22,927 [63]
Purchasing power parity ranking comparison (per capita of GDP)	15	91	115

Source: OECD,The World Economic Forum, Business Tech, World Bank, ILO, Transparency International, World Bank [162–174]

Rio Tinto’s 83% foreign-owned Australian operations account for approximately half the corporation’s global assets through the production of iron ore, bauxite, uranium, aluminium, diamonds and salt from more than 30 operating sites and processing plants. Rio Tinto ranks third in the top 2000 companies in Australia [73]. In Southern Africa, it mines uranium at Rössing in Namibia and mineral sands at Richards Bay in South Africa.

Rio Tinto’s political practices in the selected jurisdictions include tax minimisation practices, lobbying government directly, or via industry representative bodies, and collaborating with and funding the university sector to conduct research.

#### Taxation practices

Of the US $5.1 billion tax paid globally in 2017, US$3.8 billion was paid in Australia, including US$1877 million in corporate income taxes and US$1644 in royalties [48]. This compares with US$93 million in South Africa, including US$81 million in corporate taxes, US11 million in royalties and US$22 million in payroll tax. In Namibia a total of US$7 million was paid: US$6 million in corporate taxes and US$1 million in royalties [48]. A 2011 report for the Centre for Research on Multinational Corporations stated that Rio Tinto is transparent with regard to taxes and other contributions to the Namibian government from its majority owned company Rössing Uranium and, of four uranium mining corporations operating in Africa, it provided the most transparency on taxes, royalties, and other financial contributions [74]. In 2017 the Group’s effective tax rate on underlying earnings was 28.2% [48].

The Australian Taxation Office is auditing Rio Tinto regarding profit shifting through marketing hubs in Singapore which imposed a taxation rate of 5 % on Rio Tinto’s $790 million profit in 2014 [75]. Tax reductions facilitated by lobbying governments for mining-related tax deductions, together with legally available taxation-minimisation strategies, highlights the disparity between the economic obligations of individual taxpayers and those of corporations [76]. While it is difficult to quantify the level of taxation avoided by TNCs including Rio Tinto, it is sufficient to warrant Australian Taxation Office concerns [77].

#### Lobbying governments

Over the last decade Australian mining lobby groups raised revenue of $541,275,884 including $203,594,120 by the MCA [78]. Lobbying by Rio Tinto and BHP Billiton in Australia between 2007 and 2016 averted an inquiry into supply and demand in the $75 billion iron ore industry, especially concentration of market power in two very large companies [79]. Rio Tinto also lobbied to maintain discounted diesel fuel for mining companies [80] and the mining industry successfully lobbied against the Australian Minerals Resource Rent Tax (‘mining tax’) on extractive industry ‘super profits’ [81, 82] which was repealed by government [78]. Tax concessions allowable on the lobbying expenses paid by the mining industry from 2007 to 2016 represented a loss to Australian taxpayers of $162.4 million [78]. Rio Tinto’s policy-related submissions include promoting workforce ‘flexibility’ [83], and support for fossil fuels [84].

No information was available on direct lobbying by Rio Tinto in South Africa or Namibia which may be actioned indirectly through industry bodies.

#### University collaboration and funding

Rio Tinto is involved in a number of Australian research partnerships and university scholarships. The corporation’s educational portal SMART supports secondary teaching and learning in maths, science, business studies and geography [85]. The increasingly close relationship between fossil fuel industries and universities highlights the potential for conflicts of interest [86, 87]. In 1999 the MCA established the Minerals Tertiary Education Council (MTEC), which aims to provide greater input into course materials [86]. Rio Tinto also offers Australian university scholarships, and bursaries and internships at South Africa’s major tertiary institutions [88]. Universities can vary significantly in their approaches to management of competing interests [89], raising concerns over institutional decision-making [87], academic freedom [86] and rigorous use of research. A review of literature on community funding by Rio Tinto reveals an apparent preference for projects supporting business and education partnerships [90] and ecological or zoological studies [91] rather than for preventing or lessening negative environmental or social impacts from their operations.

### Major health impacts of Rio Tinto operations

Here we summarise (see Table 2) key positive and negative aspects of Rio Tinto’s operations in respect of health and /or health equity in relation to workforce, social, environmental and economic conditions within Australia and Southern Africa. Civil society respondents gave particular emphasis to issues including the limitations of the broader regulatory environment, employment, and impacts on health, social, and environmental conditions, and these views are included in the four domains defined in level 3 of the CHIA framework.

**Table 2:** Summary Rio Tinto’s impacts on social determinants of health and equity in Australia and Southern Africa

CHIA domain	Positive aspects	Negative aspects
Political and business practices	*Supply chain management* Requirements imposed by Rio Tinto on its global supply chain are likely to have positive health impacts for workers	*Political lobbying* Membership of representative organisations to influence public policy in ways unfavourable to health equity, while protecting corporate image
Workforce and working conditions	*Provision of direct employment under decent working conditions* Low, and falling rates of illness and injury for direct employees A global ‘whistleblower’ program is available to workers and all stakeholders [stet]: Australia has a high proportion of Rio Tinto’s global workforce, with an inclusive approach for Indigenous and female workers. Southern Africa: Richards Bay Minerals is a major South African employer.	*Increase in precarious, lower-paid working conditions due to practices of contracting labour through third-party organisations* Legal responsibility for workers is reduced Adverse health impacts on contract workers not ‘visible’ in corporate reporting mechanisms. Increased worker incentives to work unsafely Complex corporate structure may impede union efforts to mediate negative working conditions. Australia: Most Australian workers are contracted through labour hire organisations. FIFO operations may have negative impacts on health and well-being Southern Africa: Contract workers lack the benefits of direct employees. Relatively worse working conditions for contracted workers in Southern Africa likely to have worse health impacts than for contracted workers in Australia, eg occupational injury rates. Recourse to legal measures to seek compensation for adverse impacts weaker than in Southern Africa
Social conditions	*Rio Tinto provides some benefits to affected communities* Australia: Increased local Aboriginal participation in the Rio Tinto workforce Southern Africa: Richards Bay Minerals supports infrastructure, local procurement, skills and enterprise development, and joint ventures to facilitate skills transfer. The Rössing Foundation undertakes a broad range of community development activities.	*Rio Tinto operations impact negatively in mining localities* Australia: Relatively high miners’ wages can increase prices for goods and services in local communities. Negative community impacts from noise and air pollution from coal mining Psychological distress resulting from social disruption and environmental damage. Southern Africa: Migrant workers disrupt communities in vicinity of mining operations
Environmental conditions	*Commitment to upholding sustainable development principles* Rio Tinto signed up to International Council on Mining and Metals principles for sustainable development Disclosures under the Global Reporting Index may support the Paris Agreement to reduce global warming Australia: Commitment made to progressively return the area of the Ranger Uranium mine to a viable ecosystem under government supervision. Southern AfricaRichards Bay Minerals monitors emissions for air quality. Rossing Uranium monitors dust levels on site and in nearby town.	*Environmental risk to health and lack of remediation* Failure to remediate large final voids leaves a negative environmental legacy. Divestment may result in avoidance of responsibility for site remediation or adverse impacts of environmental damage Australia: Aboriginal community concerns over negative impacts from spills, and breaches of licence conditions at the Ranger uranium mine. Southern AfricaWorkers at Rossing exposed to dust and radon gas. Air pollution, biodiversity loss and soil contamination at Richards Bay Minerals
Economic conditions	*Contribution to national and local economies* Rio Tinto’s direct global economic contribution is made through payments to workers, suppliers, governments and community development programs. Australia: A high proportion of global taxation is paid in Australia Southern Africa: Richards Bay Minerals is the largest taxpayer in KwaZuluNatal.	*Business strategies impact on revenue for social investment* Profit shifting and/or tax reduction strategies reduce government revenues available for health and social investment. Economic costs of damage to environments and/or health are externalised to states and communities. Australia: Lobbying by the mining industry against the ‘mining tax’ and for fuel subsidies results in loss of government revenue available for public good purposes.

### Workforce and working conditions

Employment is a critical determinant of health. While secure, safe, adequately-paid work is positive for health, insecure, lower-status work can have adverse health impacts [92]. Our data examined the comparative employment conditions across the jurisdictions; information on wages and conditions and work health and safety. Some positive aspects include the thousands of jobs provided by Rio Tinto across Australia, South Africa and Namibia [93]. A Namibian activist noted that Rio Tinto employs many non-skilled labourers at the Rössing site who would otherwise have difficulty in gaining employment. In 2016 Rio Tinto employed approximately 950 workers at Rössing of whom 78.2% were men formerly disadvantaged by apartheid policies [94]. Rio Tinto is also one of the largest private sector employers of Indigenous Australians, with over 1431 full time Indigenous employees in 2017 [93]. The corporation also states its commitment to gender equity [95]. Positive workforce issues noted by Australian respondents included a high quality graduate program, redundancy package funding, and improved rosters for ‘fly in fly out’ (FIFO) workers. FIFO employment in Australia can place a heavy strain on workers and their families, with potential adverse impacts on health and wellbeing [96].

However, these positive aspects are curtailed by high levels of casual contractors, with less favourable employment conditions affecting an estimated 70% of Rio Tinto’s global workforce [97]. Industriall Union argues that Rio Tinto is not transparent about the extent to which it employs precarious workers and does not disclose how many people work on sites; reporting only direct employees [97]. Many of these precarious workers are in casual and temporary roles, and are employed by labour hire firms or are self-employed [97].

Casual contractors are employed in Australia, South Africa and Namibia, with Rio Tinto now only using contract labour for its Australian iron ore operations [98]. The National Union of Mineworkers (NUM) reports that precarious workers outnumber permanent workers at Rio Tinto’s Richards Bay Minerals operation [99].

In 2015 the Mineworkers Union of Namibia delivered Rio Tinto’s Rössing uranium mine management a petition demanding an end to the exploitation of contractors, which included paying some contractors only one-seventh the wage of regular workers, forcing them to work longer hours with less job security, and claimed victimisation of union members [100–102]. The union has also reported concerns over violations of sub-contractors’ right to freedom of association and right to collective bargaining [99]. A Namibian respondent explained:

*Yes they have contract workers and the contract workers are dependent on the contractor … Rössing has not got the same obligations to contract workers as to their permanent workers and that makes it very difficult. They get exchanged quickly, they haven’t got medical aid, they haven’t got a pension fund and the Rössing workers have that.*

Industriall Union’s 2014 global campaign against precarious work practices by Rio Tinto in Namibia argued that the company refused to address the concerns of the workers via their union representatives; did not comply with the Labour Act /Agreement that prohibits employers from unilaterally changing conditions of employment; and that they negotiated in bad faith by withholding critical information during negotiations [103]. Rio Tinto has an elaborate corporate structure with shifting responsibilities which can impede union efforts to mediate precarious employment conditions, including the rise of contract labour [97].

Job insecurity associated with contract or precarious forms of employment has both financial and mental health consequences [104], and can make workers more reluctant to use sick leave [105]. This was a concern raised by one Australian union respondent about precarious workers in one of Rio Tinto’s operations, who contended ‘They are less likely to report unsafe situations and far less likely to take time off when they are sick or injured because they don’t get paid for it’. Other potential problems include loss of expertise through loss of older workers, including occupational health and safety knowledge, leading to increased exposure to hazardous substances and other risks from ‘corner cutting’ [105]. One Australian union representative was also concerned at the lack of transparency in reporting Rio Tinto’s growing proportion of sub-contractors compared with directly-employed workers, claiming that this ‘creates two quite distinct classes of workers in the mining industry’, thereby highlighting implications for health equity. Another Australian union representative cited the lack of guaranteed hours, lower superannuation payments, lack of reimbursement for travelling time, and easier dismissal of workers through third party sub-contracting arrangements. People who are unemployed or lack job security consistently report the lowest levels of subjective well-being and self-rated health [106], and are at greater risk of mental health problems [107].

In a 2012 submission to a government review of Australian labour law Rio Tinto declared that it is ‘committed to establishing a direct relationship with every employee irrespective of the employment arrangement under which the employee has been employed’ [108]. Rio Tinto’s preference for individual contracts over union collective agreements has led to the de-unionisation of much of its Australian workforce [109]. This was a cross-jurisdictional concern raised by a South African respondent:

*The question around contract labour becomes even more critical because that fundamentally undermines the foothold of organised labour. Organised labour can be a very powerful voice for both occupational health and safety and for community impacts if people are living in the area around the mines… And, also even though we may be upper middle income we are one of the countries with the greatest Gini Coefficient, so inequality inside our country. So, that also very much shapes the dynamic in the society…If you're sub-contracted then you're more vulnerable. You're certainly more vulnerable in terms of occupational health and safety*.

Research by Farber et al. (2018) highlights the critical role of strong trade unions in mediating poor working conditions and inequality. These researchers found that over the last nine decades, when unions expand at either the national or state level, they tend to attract the membership of unskilled workers and increase their relative wages, thereby helping to address inequality [110]. Conversely, where so-called ‘right-to-work’ legislation has undermined the right to organise, occupational mortality has risen, illustrating the protective effect of unions on workplace safety [111].

Issues of occupational health and safety have been raised in respect of uranium mining. Rössing in Namibia is the fifth largest open pit uranium mine in the world. Earthlife Namibia and Commission for Independent Research and Information about Radiation (CRIIRAD) have monitored areas located near uranium mines in Namibia, especially Rössing. The dose rate measured in 2011 by CRIIRAD on the parking area of the Rössing mine was approximately six times higher than natural background value [112]. A Namibian activist highlighted the associated negative aspects of employment in the uranium industry:

*They work eight hours in this toxic environment. There is such a lot of dust. They are blasting and they are moving and they are crushing and milling, so everywhere is dust. This dust is full of toxic particles and full of radioactive particles and we have background radiation.*

In a 2014 study carried out with present and former Rössing workers, 39 of the 44 respondents complained of health problems and difficult working conditions causing back pain, breathing, hearing and visual problems. The biggest concern however was constant dust exposure. Most workers also stated they were not informed about their health conditions or exposure to radiation. While some workers consulted a private doctor for a second opinion, this is not an option that most workers can afford [113].

#### Work, health and safety regulations across jurisdictions

Our data suggest there are significant difference between work, health and safety regulations in Australia and Southern Africa. In Australia *Safe Work Australia* is the the national work, health and safety policy agency, with regulation governed at the state and territory level under 10 separate statutes, with some variations in legal requirements and enforcement. All Australian statutes grant inspectors broad powers; including to issue improvement and prohibition notices and to prosecute those found in breach of legislation [114]. The International Labour Organisation (ILO) reports that in contrast, South Africa accident compensation may take years, or may even never eventuate [115]. In South Africa workers are mainly protected under the Occupational Health and Safety (OHSA) Act and the Mines Health and Safety (MHSA) Act [116]. The OHSA Act covers the non-mining sector whereas MHSA covers all mining; but neither deals with workers’ compensation. In South Africa compensation is governed by the Compensation for Occupational Injuries and Diseases Act (for non-mining) and Occupational Diseases in Mines and Works Act (for mining).

Due to enormous backlogs and lack of efficiency in both compensation systems the current trend is civil litigation. This has culminated, for example, in recent legal settlements including US $400 million for tens of thousands of South African gold miners who had contracted silicosis [117]. Therefore, workers in both mining and non-mining arguably get poor service for compensation in both systems and the effectiveness of the MHSA as a preventive tool for workers in the mining sector is debatable [118, 119]. Industriall Union claims gross violation of health and safety regulation for outsourced workers at Richards Bay Minerals [97].

Exact comparisons between jurisdictions are difficult due to factors including the type of mineral produced and the level of mechanisation, but workplace fatalities in South Africa’s mining industry are four times higher than those in Australia [115]. A report by the NUM stated that by 2013 the fatality rate in South Africa was 0,09 deaths per million hours worked. While that is a steep improvement from the 0,32 of 2003, it’s still ‘not a patch’ on Australia’s 0,02 deaths per million hours [120]. Rio Tinto does not report work, health and safety statistics nationally, only globally, and at the local operational level. In 2017 it reported both a safety-related and a health-related fatality at its managed operations in Utah and Western Australia respectively [43]. Three fatalities occurred at the operations of non-managed joint arrangements in Brazil, Papua and Mozambique in 2017 [43].

Rio Tinto states that there was a 43% decrease in the rate of new cases of occupational illness per 10,000 employees annually compared with 2016, and the all injuries frequency rate (AIFR) per 200,000 h worked improved by 5% from 2016 [43]. Rio Tinto explains that their data relating to the all injury frequency rate includes all employee and contractor exposure hours and incidents, but that new cases of occupational illness are reported for employees only [43]. However, self-reporting has serious limitations and independent monitoring and auditing would be preferable.

### Social conditions

Impacts of extractive industry operations on social conditions include the effects on local goods and services and local community life. Rio Tinto’s Communities and Social Performance standards guide community relationships [121]. The corporation’s own literature claims an extensive range of positive social contributions in its Australian, Southern African and Namibian operations, including community development, educational, and Indigenous support projects [93]. One indicative community support program is the funding by Rio Tinto’s Western Australian Pilbara iron ore operation for educational initiatives including science, technology, engineering and maths (STEM), school readiness, and to improve educational attainment in Indigenous populations, with a particular focus on the Pilbara Aboriginal community. The other key focus is enhancing community vibrancy through arts, culture and sport as part of Au$17.9 million to 130 community programmes and $9.8 million in-kind support in Western Australia [122].

Several activists discussed health and social conditions associated with coal mining in eastern Australia. One union representative spoke positively of Rio Tinto’s Australian contribution:

*I think in general economic terms, Rio Tinto’s involvement in Australia has been generally positive. In some instances there have certainly been very beneficial impacts upon the economic and social health of some of the communities in which they have operated.*

A South African respondent noted positive social impacts from Rio Tinto’s investment in the local economy by engaging local service providers for site rehabilitation and cleaning services. Another stated that Richards Bay Minerals support bulk water supply for the local municipality as part of their social labour plan (SLP) commitment. Rio Tinto advises that in 2015 Rössing committed N$18 million (US$1,302,000) towards implementing community initiatives. The focus was on improved primary and secondary education through the implementation of learner and teacher support programmes, vocational skills development, scholarships, apprenticeships and local economic diversification. Other financial initiatives included minor contributions such as personal health awareness, biodiversity protection through support of the annual birdwatching event; and waste management activities at local primary and secondary schools [123].

Negative aspects of Rio Tinto’s operations in Australia include the social upheaval on both Indigenous and other Australians from the impact of the Ranger Uranium mine [124], and communities in New South Wales impacted by coal dust, noise, poor infrastructure maintenance, and some even being forced out of their homes, as an activist explained:

*Solastalgia is the term, and he [psychologist] thinks it’s suffered by people and that’s what we’re feeling about [our town]. [Respondent reads aloud…] ‘Solastalgia is an emplaced or existential melancholia. It’s a negative transformation that is a desolation of a loved home environment’. That’s the way we’re feeling about our home.*

One activist spoke of their efforts to be well-informed about Rio Tinto’s operations in order to advocate for the community; despite a strong sense of powerlessness:

*We’ve become experts in fields that we never dreamt of being experts in…We know that we are in the right but the dealings of these multinational companies, they’re so powerful with the government and we just see that – you get that feeling that you just don’t matter. You feel like collateral damage basically.*

Other participants articulated more specific negative health and social conditions linked to Rio Tinto’s Australian coal mining operations:

*We sleep with earplugs here. Our house is very, very dusty…so we are living in a very dusty environment…Our area has one of the highest asthma rates for 9-15 year olds in the state. It is pretty bad. We have dust monitors around. We often have toxic fumes go up after a blast.*

Combining the research traditions of the sociology of mental health and environmental inequality studies, Downey and van Willegan (2005) examined the impact of industrial activity on individual well-being and found that it is associated with perceptions of individual powerlessness and neighborhood disorder, leading to higher levels of psychological distress [125, 126]. Concepts of power, autonomy and control in exercising choice are important factors in gaining access to resources to promote and maintain health [127]. Health inequalities are caused by the unequal distribution of power, as well as goods and services, both globally and nationally [128]. One respondent living with the health impacts of coal mining in Australia spoke of adverse mental health impacts, including feelings of grief and loss and description of the ‘resettlement effect’ [26]:

*You feel like you have a lot of the control of your life lost, and feeling of not belonging, so there’s no attachment any more. You feel the loss of time and effort and energy and health, the environment and its opportunities. You sort of feel like your future has been stolen… forced out of our home…It’s just a big sense of loss and grief.*

In Namibia, uranium workers housed in the township of Arandis have historically been exposed to dust, and radon gas with documented health impacts, especially an increased risk of lung cancer [113, 129].

### Environmental conditions

Rio Tinto states that it supports the Paris Agreement to reduce global warming, and research into carbon capture and storage and low emissions technologies. It provides extensive information on maintaining sustainable operations and notes that it aims for substantial decarbonisation by 2050 [93]. However, adverse environmental impacts are documented in a Global Atlas of Rio Tinto’s site operations [130]. These include soil contamination, groundwater pollution or depletion, and mine tailings spills at Rössing in Namibia; and air pollution, biodiversity loss, soil contamination and erosion, waste overflow, deforestation and loss of vegetation cover and mine tailings spills at Richards Bay in South Africa [131].

Richards Bay Minerals is one of four extractives industries that consume 60% of the available water supplied in the Mhalatuzi district in KwaZulu Natal province. In 2015 it was reported that residents were facing water restrictions due to drought conditions and the high water use by the mining industry at Richards Bay. A civil society activist in Namibia explained that the Rössing uranium mine also uses scarce water and electricity resources which affects the local community, and country as a whole:

*They use a lot of water in a water scarce country. They demand one third of Namibia’s total water usage and that’s a lot one third. That’s the same with electricity. Then there is ground water contamination which is very, very serious and the ground water is flowing very slowly so it did not reach the coast. The mine is roundabout 60 kilometres east of the coast but there is a lot of east wind. They have strong east winds from time to time and then the dust, the particles, are blown by the wind to the coastline and there are lots of towns.*

Despite this, residents were concerned that forced mining closure would have devastating social and economic consequences [132].

In Australia the National Pollution Inventory highlights the high level of pollution associated with coal mining in the Hunter Valley New South Wales [133]. More than 30,000 people live within 500 m of coal rail corridors, stockpiles or loading facilities in the greater Hunter region. Exposure to airborne dust may trigger asthma attacks and allergic reasons, contribute to breathing-related problems and cardiovascular disease and a reduced life span [134, 135].

Rio Tinto’s corporate literature reports contamination of ground water in the Northern Territory at the Gove Alumina refinery [136], and the Public Health Association of Australia has reported on the high number of significant environmental safety incidents at the Energy Resources Australia’s Ranger Uranium Northern Territory operations [137]. Rio Tinto states that ‘We progressively rehabilitate to the extent practicable’ [93], but a key issue for civil society actors in both Australia and Southern Africa is the lack of site rehabilitation, especially in respect of the massive ‘voids’ left by open cut mines. An Australian respondent with an interest in mining practices argued in respect of mining companies more generally:

*Mining companies, by and large, will defer any cost that they can from today until tomorrow. And in order to relinquish land it’s a significant cost. So they’re deferring and deferring and deferring for later, and the mine managers of the day have no incentive to do otherwise. It is not in their brief.*

An activist campaigning against the negative impacts of the Rio Tinto coal mining operations stated:

*It’s not just the [named mine]. It is the whole area, the dust, the trees. They have a different look about them. They’re choking. It’s just an appalling – the fact that we have no policy on final voids and rehabilitation.*

Another concurred:

*You have no idea what these places [voids] look like. They’re hundreds of metres deep; 10 kilometres by 5 kilometres hole in the ground…They put them into what is called care and maintenance which means they don’t actually close, that so that means they don’t actually have to rehabilitate.*

A similar concern was raised by a Namibian activist over the environmental impacts from mining low-yield uranium, and long-term implications for health:

*Last year Rössing moved 14 to 15 million tonnes of rock and earth to produce one tonne of uranium. You can imagine how big the hole is and how big the waste dump is, how big the tailings is, how much water they use and how much electricity they use. All this is a danger for Namibia…[The tailings are] a catastrophe because it’s a huge area which gets contaminated and that sinks into the ground, into the ground water eventually. Also there is always seepage….What lining will last for 200,000 years and who can maintain such a thing?*

Other environmental concerns cited by respondents in Australia included uranium tailing dams spills, the lack of action on mitigating the impacts of climate change, the threat to Aboriginal artefacts, destruction of critical areas for endangered species habitat, and the lack of effective regulatory regimes to deal with hydraulic rock fracturing [‘fracking’], air and water pollution, and other systemic problems. An Australian activist made suggestions for strengthening the regulatory landscape to deal with these problems, arguing:

*We need next-generation environmental laws, imposing far stricter controls in relation to all forms of pollution and the protection of biodiversity. We need a ban on the opening of new coal mines and other fossil fuel resources and a rapid but fair transition out of existing fossil fuel mining operations.*

### Economic conditions

Rio Tinto’s operations have a significant impact on national and local economies and public revenue in the countries in which they operate [138]. In Australia the potential public benefits of the corporation’s economic investment has been negatively affected by successful lobbying by mining industry bodies and corporations to promote a regulatory environment conducive to business interests [80], and through its strategies to reduce corporate taxation. For example, lobbying by Rio Tinto and BHP Billiton to maintain cheap diesel for mining companies costs Australian taxpayers over $4.5 billion per year. The estimated loss from overturning the Minerals Resource Rent Tax was $5.3 billion over the forward estimates [78]. An Australian Senate inquiry found that Rio Tinto’s Singapore hub made a $790 million profit but paid a 5 % tax rate in 2014 [139]. Adverse economic impacts also result from the reduction in Rio Tinto’s global workforce associated with the end of the ‘mining boom’. This included a reduction of 1650 Australian workers in 2016 as part of 4750 Australian jobs lost since 2012 [140].

Other negative economic aspects include externalising environmental costs onto Australian, South African, and Namibian communities through a range of strategies [130, 141, 142]. For example, in 2015, the New South Wales government acknowledged that it would cost $2 billion to remediate one specific void from Rio Tinto’s coal mining operations, but this has not eventuated; leaving a negative legacy for the wider community [143]. A South African respondent cited economic stress from a ‘boom and bust’ cycle which has resulted in scaling down of operations and loss of employment at Richards Bay Minerals. However, Rio Tinto states that while challenges remain, particularly in the area of low market prices which may persist for several years, the longer-term outlook for the nuclear industry remains positive [94].

#### The role of regulation

Evidence indicates that strong national (and sub-national) regulation is required to mediate the health impacts of TNCs [144] on health and equity. Australian activists monitoring Rio Tinto’s coal mining operations spoke of their ‘battle’ against the power of both Rio Tinto and government:

*In this battle along the way over the past eight years, we actually ended up in the Land and Environment Court where we won our case hands down. They [Rio Tinto] got thrown out of court. They applied again. They appealed so we went to the Supreme Court of Appeal where we won again. So then after that win, the government changed the goal post to suit Rio Tinto. When they appealed, they appealed the findings that they took away our right of merit appeal so we were left - even though we were winners in court, we were losers because they took away our right of appeal.*

An Australian union representative stated:

*The problem is as much with the governments – be they national, state or local – and their enforcement of their own environmental guidelines, and in some cases, a lack of environmental guidelines that allows mining companies to do whatever they want and walk away from the damage they have undertaken*.

One Australian respondent shared a commonly held view on the need for greater regulation, and reflected on Rio Tinto’s political practice of using a peak body to advance policy positions not in keeping with its preferred corporate image:

*The existing regime of mining law in Australia is entirely inadequate to the job of protecting the environment…and there’s just the general problem of corporations having too much power both in terms of how their participation in the political process is treated, the kind of tax breaks that are given to their representative peak body... So one of the tricks Rio Tinto [and other mining corporations] has played over the years, is they’ll take a corporate view on one thing that might be progressive but will then be paying money to a peak body… which will be pursuing some sort of awful troglodyte line on something, whether it’s Indigenous affairs, the environment or climate change.*

Other regulatory concerns raised by respondents included a lack of effective grievance mechanisms for South African mining companies as a result of under-resourcing of government inspectorates, and a lack of parity between Rio Tinto’s global operations by the limiting of action to minimum standards in each jurisdiction of operations. A South African respondent explained that mining company staff do not have the training or expertise to comply with legislation and that local municipalities lack resources, resulting in deficits in planning and services:

*In South Africa we have probably the most advanced legislation in this area [social and labour plans]. However, it sounds good on paper. However, in practice there are some really big problems. One of the problems would be the expertise inside a company to be able to drive this process efficiently, to understand what needs to be done and how do you consult - you're required to consult with the municipality and the community, and how do you do that successfully? Then, the local municipalities themselves are under-capacitated, so they don't have often the necessary planning skills and there are big deficits within their own expertise, and sometimes capacity to deliver on basic services.*

In Australia concerns were also raised about the lack of enforcement of policies and regulations; especially in respect of environmental concerns. In reflecting on the multitude of concerns one Australian respondent from a large NGO argued:

*Ideally, there would be a change to the DNA of business corporations so that instead of just being responsible to shareholders that they also owed fiduciary obligations to the communities around them and to the environment. We need climate triggers in relation to all new developments. We need a raft of laws around shifting obligations back on to the powerful and that’s in relation to labour but also in relation to other areas.*

## Discussion

Here we present some general reflections on Rio Tinto’s governance practices and performance related to health and health equity, compare performance across jurisdictions, and consider the influence of national and international governance frameworks.

Rio Tinto generally operates in accord with the regulatory requirements of the three selected jurisdictions. It also commits to corporate social responsibility in a range of areas, which are positive for health. These include applying a code of conduct for companies operating in Rio Tinto’s supply chain, and a range of activities under its Communities and Social performance standards. However, specific corporate social responsibility initiatives may be undermined by financial accounting practices [145], or alliances with industry bodies promoting policies antithetical to population health [78]. Furthermore, our comparative assessment indicates that Rio Tinto generally does just what is needed to meet regulatory requirements in the countries where it operates, rather than apply consistent corporate standards, meaning that practices related to health are generally of a lower standard in both South Africa and Namibia compared to Australia, because regulatory frameworks are limited or not as well enforced or resourced, or union support is weaker.

Two key areas where we found decidedly mixed performance for health were in employment, and environmental performance. Employment is an important determinant of health. Rio Tinto is a major employer in Australia and a significant employer in Southern Africa, and in both cases positively addresses equity by employing people from groups with otherwise limited opportunity. However, the apparent corporate strategy to shift major elements of its workforce in all selected jurisdictions to third-party contract arrangements is a significant negative for health and likely to create a gap between groups of workers in terms of remuneration, job security and/or working conditions. This represents a transfer of corporate responsibility to labour hire companies, individual workers, subsidiary companies, and supply chains [146].

Adverse health impacts on contracted Rio Tinto workers, including after they cease employment, may be overlooked in corporate self-assessment or reporting. Again, the negative implications of this gap for disadvantaged workers are likely to be generally worse for workers in South Africa and Namibia. Here the power of workers to achieve terms and conditions of work conducive to health, or seek compensation is relatively worse, and these processes play out against a background of greater social and economic inequality.

The fact that Rio Tinto is a signatory to agreements on climate change is positive, although it is difficult to judge its performance in this area from the evidence we gathered. However, our research indicates a range of adverse impacts both in terms of long-term, un-remediated damage to environments in the vicinity of mine sites and associated damage to physical and psychological health for communities living in those areas. Corporate strategies of ‘maintaining’ rather than closing mines where production has ceased, or sale of assets to other companies provide a means to avoid liabilities for site remediation. Again, the effect of such strategies can be to shift costs to governments (and taxpayers) and communities. Also, inconsistencies have been found between how companies, including Rio Tinto, rank their application of widely-used sustainability guidelines and publicly available information that is used for verification [147]. We did not identify any evidence to suggest environmental performance is markedly worse or better in any of the jurisdictions studied – there are significant problems in all three countries.

In economic and political terms, Rio Tinto’s contributions to economic activity and government revenues within the three selected jurisdictions have the potential for broader benefits for health, by stimulating wider employment opportunities and/or providing revenues for government expenditure on social determinants of health such as education and primary health care. However, Rio Tinto’s political and business practices also raise concerns, especially it relation to strategies to shift profits and reduce taxes paid, and use of lobby groups as a front to influence government policies and pursue commercial interests [78] while also protecting the corporate image. Potential conflicts of interest in relation to university funding and the level of corporate influence over government policy pose serious threats to health.

Our research highlights that actual and /or potential conflicts of interest occurred within the context of lobbying governments and regulatory bodies, and from partnerships with universities. Codes of conduct are necessary in both the public the private sector to combat malfeasance and corporate fraud [148]. Several international organisations, including the United Nations Convention Against Corruption (UNCAC), have developed guidelines and devised protocols to assist standardising definitions and adopting preventive and enforcement mechanisms [148]. Governments should adopt clear policy/guideline/legal frameworks for preventing, detecting and managing conflicts of interest to ensure both the causes and effects are properly addressed [148].

One avenue for aiding transparency and avoiding conflicts of interest in extractive industries would be to mandate that annual reports include a section on the health and welfare of the workers and communities adjacent to work sites compiled by independent civil society actors without any conflict of interest. This reporting would both augment and contextualise corporations’ documentation of the social, economic and health impacts of their products and operations.

### Policies that may assist in making extractive industry’s operations more health promoting

In assessing both the benefits and negative impacts that TNCs generate, there are ways in which business can be conducted with fewer health impacts (See Table 3). For example, TNCs should be required to pay tax at the point of profit generation and thereby contribute to that country’s public good. Restrictions should be imposed on the ability of corporations to lobby governments and unduly influence the democratic process. More attention should be given to employment conditions which are being eroded by increased sub-contracting and the impact of ‘Fly in fly out’ operations on families and communities. Rio Tinto’s practice of ‘direct engagement’ with workers arguably undermines the recognised role of unions in protecting workers’ best interests. Stringent environmental controls should be mandated and monitored by government in respect of both compliance and the level of impact on local communities.

**Table 3:** Recommendations for improved regulation of extractive industries

• Adopt clear government policy/guideline/legal framework on preventing, detecting and managing conflict of interest	
• Develop an international agreement to ensure that all taxes are paid in country of profit generation	
• Cease inequitable taxpayer funded extractive industry subsidies	
• Restrict government lobbying undermining the democratic process	
• Halt the erosion of employment conditions through contracting	
• Mandate greater environmental controls including reduction in greenhouse gas emissions	
• Counterbalance fiduciary duty to shareholders with social obligations to local communities	
• Prevent the use of voluntary codes to undermine or prevent enactment of legislation	
• Develop an independent auditing process to ensure that TNCs comply with their stated commitments	
• Endorse the proposed UN Binding Treaty on Business and Human Rights	

### Research limitations

A major limitation of our research was the non-participation of Rio Tinto or industry representatives in the interviews. While this limited our access to an industry view on the health impact of their operations, the research shows that it is possible to obtain sufficient salient material on TNCs from extensive publicly available sources, as identified in our study on McDonald’s in Australia [10, 34] and in earlier research by others on Walmart [149]. As Rio Tinto uses professional image management to prevent and manage risk [150], this may account for their decision to not engage. Understood through a power lens this is just one of the ways by which powerful actors can influence research [151]. Other limitations were that there was no specific policy, proposal or decision point the CHIA process was trying to influence, nor an explicit advocacy process, nor was it community-led. Therefore, processes concerning weighting of evidence, engagement, governance structures, and managing conflict or disagreement did not need to be established.

## Conclusion

This paper demonstrates that it is possible to identify potential and actual health impacts of an extractive industry TNC operating across different jurisdictions. It allows for an overall understanding on the health impacts, based on assessing corporate literature and with additional data collection from key informants. Identifying the positive and negative health impacts from Rio Tinto’s operations by utilising the CHIA framework may potentially assist governments and international bodies to devise appropriate regulatory frameworks, and provide TNCs with insights to augment corporate social responsibility initiatives, enhance their social licence to operate, and provide decision-making support. Civil society actors and trade unions may benefit from an increased evidence base by which to inform their advocacy towards improved social and health investment and equity outcomes [10].

This research highlights the need for strong regulatory frameworks to help support positive health impacts and avoid or mitigate negative impacts from corporate operations. If Rio Tinto and other TNCs were also mandated to make reparation for externalising the economic, social and environmental costs of their operations the situation could be very different.

Civil society will continue to play a role in advocating for necessary change, as will the actions of interventionist governments. Existing voluntary codes should be seen as adjuncts to properly enforced protective regulations, not as alternatives. Under the UN Global Compact business actors represent the majority of participants, with civil society organisations taking a secondary role in assisting corporations meet established goals and maintain code legitimacy. This raises questions concerning the relative power of civil society in global governance more generally. On the other hand, the UN work developing a Binding Treaty on Business and Human Rights affords much greater civil society input to the work of the open-ended intergovernmental working group (OEIGWG) on transnational corporations and other business enterprises with respect to human rights [152]. As a result, the elements being developed for this legally binding instrument offer greater enforcement capacity and meaning [153, 154].

The Binding Treaty has its genesis in the UN Norms on Transnational Corporations and Other Business Enterprises which sought to impose on companies the same duties as states to promote, secure and respect human rights. This was followed by The UN Guiding Principles on Business and Human Rights, or guidelines for states and businesses to prevent, address and remedy human rights abuses committed in business operations [155]. In 2015 the UN Working Group on Business and Human rights negotiated to develop and mandate an international legally Binding Treaty to respond to the inherent limitations of guiding principles and other voluntary initiatives [156]. This remains a work in progress.

Extractive industry and other TNCs will continue to be an important part of the global economic system, if only because complex societies need the logistics and efficiencies they can deliver. However, there is a crucial and legitimate role for public health advocates and researchers in posing questions about the extent of their power and the way that this can undermine population health [157]. Despite undertaking corporate social responsibility initiatives the corporate entity ultimately functions to serve its own interests, and has no real alternative when confronted with an option between profit and social good [158]. Global companies have ‘taken on the mantle’ of central organisers of the global economy, in addition to national economies. They determine ‘who gets what’, but remain relatively understudied compared with state and society [159]. Our research augments this literature.

## Abbreviations

BCA: Business Council of Australia
CHIA: Corporate Health Impact Assessment
CRIIRAD: Commission for Independent Research and Information on Radioactivity
FIFO: Fly in Fly Out (workers)
FPIC: Free, Prior and Informed Consent
GDP: Gross Domestic Product
HIA: Health Impact Assessment
ILO: International Labour Organization
MCA: Minerals Council of Australia
MHSA: Mines Health and Safety Act
MTEC: Minerals Tertiary Education Council
NUM: National Union of Mineworkers
OECD: Organisation for Economic Co-operation and Development
OEIGWG: Open Ended Inter-governmental Working Group
OHSA: Occupational Safety and Health Administration
SDH: Social Determinants of Health
TNC: Transnational corporation
UNFCCC: United Nations Framework Convention on Climate Change
